# rGO/PAN Composite Membranes Obtained In Situ Using Hydrothermal Reduction of GO in the Polymer Bulk

**DOI:** 10.3390/ma19020442

**Published:** 2026-01-22

**Authors:** Beata Fryczkowska, Łukasz Migdał, Janusz Fabia, Czesław Ślusarczyk, Ryszard Fryczkowski

**Affiliations:** Faculty of Materials, Civil and Environmental Engineering, University of Bielsko-Biala, Willowa 2, 43-309 Bielsko-Biala, Poland

**Keywords:** hydrothermal reduction, graphene, graphene oxide, composite membranes, polyacrylonitrile, nanoparticles

## Abstract

A new method of in situ hydrothermal reduction of graphene oxide (GO) to reduced graphene oxide (rGO) in polymer bulk was developed, which involves heating GO/polyacrylonitrile (PAN) composite membranes (0.5; 1.0; 2.0% *w*/*w* of GO/PAN) in the presence of water vapor at a temperature of 120 °C and a pressure of 0.2 MPa. As a result of this process, membranes containing rGO were obtained, as confirmed by FTIR, Raman, WAXS and TGA studies. The composite membranes obtained after hydrothermal reduction of GO to rGO (B60, C60, D60) were substantially different from the initial membranes containing unreduced GO (B0, C0, D0). The hydrothermal reduction process clearly influenced the physicochemical properties (reduction of apparent density, water sorption, and increase in the contact angle) and transport properties of the B60, C60, and D60 membranes (decrease in water flux by ~104 [dm^3^/m^2^ × h] and even ~348 [dm^3^/m^2^ × h] compared to the initial membranes).

## 1. Introduction

Research on methods of obtaining and using graphene has been described in publications over the last 20 years. Chemically, graphene consists of sp2-hybridized carbon atoms joined together in a flat, two-dimensional (2D) structure composed of hexagonal rings resembling a honeycomb [1,2,3]. Graphene can be obtained using various methods. The simplest method involves mechanical delamination of graphene layers from graphite using adhesive tape [4]. According to the literature, five main graphene production pathways can be distinguished: mechanical exfoliation of graphite, chemical vapor deposition (CVD), epitaxial growth on an electrically insulating surface, solvothermal synthesis and reduction of the graphene oxide (GO) [5,6,7,8,9,10]. The most widely used method of obtaining GO is chemical oxidation of graphite (exfoliation). In this reaction, oxygen functional hydroxyl, carbonyl, carboxyl and ether groups are formed between the graphite layers and on the edges [3]. These groups can be removed to obtain reduced graphene oxide (rGO). The methods of obtaining rGO include chemical, thermal, electrochemical, photochemical, microwave, hydrothermal and solvothermal reduction, and other methods [11,12].

The chemical reduction method consists in oxygen groups being removed using reducing substances such as hydrazine, hydroquinone, sodium borohydride, vitamin C, ethylenediamine or hydroiodic acid, and various reaction conditions (pH, temperature, ultrasound, etc.) [13,14]. The thermal method, on the other hand, involves rapidly heating GO to a temperature up to 1050 °C in the presence of an inert gas: argon or nitrogen [7,10,15,16]. The literature also reports that thermal reduction of GO is possible during structural studies of this material. Alotibi et al. obtained rGO in situ using a TGA instrument [17]. Other researchers carried out the reduction of graphene oxide (GO) using in situ thermal transmission electron microscopy (TEM) analysis and electron energy-loss spectroscopy (EELS) [18]. Elsanousii et al. studied an in situ approach to GO reduction induced by laser irradiation during Raman spectroscopy measurements [19]. In the solvothermal method, GO is dispersed in various organic solvents, after which the resulting dispersion is heated for a certain time at an elevated temperature. Zhu et al. described the possibility of reducing GO in propylene carbonate by bath sonication at 150 °C [20]. Other researchers used dimethyl phthalate (DMP) as a solvent and a temperature of 250 °C [10]. Li et al. obtained rGO in dimethylformamide (DMF) by heating the dispersion at 150 °C [21]. Our research group has developed a modified method for the thermal reduction of GO to rGO, which uses wet graphene oxide and a temperature of 300 °C [22].

An interesting way to reduce GO is the hydrothermal method. Ghorbani et al. heated a GO dispersion at 140 °C for 6 h under a pressure of 0.4 MPa [23]. Other researchers obtained rGO by autoclaving at 160 °C for 4 h [24] or at 100–200 °C for 24 h [25]. Still others used heating of an aqueous GO dispersion under a reflux condenser, purged with nitrogen, for 9 h [26]. The two-stage, microwave–hydrothermal method, on the other hand, involves two hours of ultrasonication of graphene oxide and then its reduction at 180–200 °C [12]. Each of the above methods produces an aqueous dispersion of reduced graphene oxide, which can be used to create new materials. The literature also describes the possibility of reducing pure GO foils using the hydrothermal method in an autoclave, at a temperature of 200 °C, for 20 h, under a pressure of 1.2 MPa [27].

For many years, our research group has been conducting studies on obtaining GO and reduced graphene oxide (rGO). In addition, we have been extensively researching the production of polymer composites with the addition of GO and rGO. As the polymer matrix, we use a widely applied engineering polymer—polyacrylonitrile (PAN). This polymer is characterized by good chemical resistance and light resistance, thermal stability, favorable mechanical properties, and thermal conductivity [28,29,30]. PAN is not a material that can be processed thermally; however, it can be processed from solution because it is soluble in many organic and inorganic solvents [31]. This polymer is widely used in many areas of human life, ranging from polymer-based structural materials to fibers (its earliest application). Owing to its excellent chemical resistance, PAN is used in membrane techniques (microfiltration, ultrafiltration, desalination, reverse osmosis, pervaporation, hemodialysis, membrane distillation, adsorption membrane) [29,30,32].

Another important feature of PAN is its ability to acquire new properties through physical or chemical modification. In our research, we focus on modifying PAN by incorporating GO or rGO. The nanofillers are incorporated (as dispersions in DMF) into a PAN solution (in DMF), followed by the formation of composite membranes using the phase inversion method. The introduction of GO as an additive is relatively straightforward due to its hydrophilic nature. In contrast, the hydrophobic properties of rGO make it difficult to obtain a stable dispersion. Therefore, we investigated the feasibility of performing in situ reduction of GO to rGO within the composite.

Based on the analysis of literature reports [12,13,24,25,27,33,34] and guided by the goal of minimizing energy consumption, we selected the hydrothermal method for reducing GO to rGO. To this end, we prepared a series of composite membranes containing 0.5, 1.0, and 2.0% wt.% GO/PAN. A simple, sealed vessel equipped with a thermometer and a valve with a manometer was constructed. The process was carried out under mild conditions: at a temperature of 120 °C, a working pressure of 0.2 MPa, and in the presence of water vapor.

**Figure 1:**
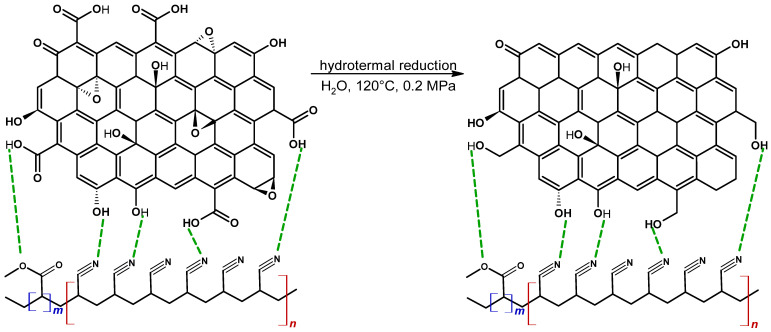
Hydrothermal reduction of GO to rGO in a PAN matrix (where *n* is ~94% acrylonitrile; *m* is ~6% methyl acrylate; green dashed line indicates hydrogen bonds).

The primary goal of our research was to test the feasibility of bulk GO reduction (Figure 1). Another goal was to select the optimal time for hydrothermal reduction of GO to rGO. As a result of our research, we obtained rGO/PAN composite membranes. Structural studies (FTIR, Raman, WAXS, TGA) allowed us to observe changes in the nanoadditive structure, confirming the in situ reduction of GO to rGO in the PAN matrix.

## 2. Materials and Methods

### 2.1. Materials

Polyacrylonitrile (PAN) (MW = 85,000): copolymer (93.9% acrylonitrile/5.8% methyl acrylate/0.3% methallyl sulfonate) was purchased from Goodfellow Cambridge Ltd., Huntingdon, UK. Graphite powder (<20 μm) was purchased from Sigma-Aldrich, Poznań, Poland. N,N-dimethylformamide (DMF), min. 95% H_2_SO_4_, 30% H_2_O_2_, and KMnO_4_ were purchased from Avantor Performance Materials Poland S.A., Gliwice, Poland.

### 2.2. GO Synthesis and Preparation of GO/DMF Dispersion

Graphene oxide (GO) was obtained according to the modified Hummers method [33]. GO synthesis and characterization (XRD, DSC, FTIR) were very similar to those obtained in our earlier work [35]. Wet graphene oxide was placed on a glass funnel with a cellulose filter and then washed five times with DMF to remove water. The dispersion obtained in this way contained 3% GO/DMF.

### 2.3. Obtaining Pure PAN Membranes and GO/PAN Composite Membranes

Membranes were formed by the wet phase inversion method. First, a 12% solution of PAN in DMF was prepared. For this purpose, the polymer was mixed with the solvent and then left on a magnetic stirrer for 24 h (sample A). Solutions necessary for the formation of composite membranes were then prepared. The appropriate amount of 3% GO/DMF dispersion was weighed and then the appropriate amount of 12% PAN/DMF solution was added to obtain solutions (B, C, D) with the subsequent GO/PAN/DMF concentrations (Table 1).

The PAN/DMF and GO/PAN/DMF solutions were poured onto a clean glass plate and spread using a casting knife with a gap width of 0.2 mm. Finally, the polymer film was rapidly coagulated in distilled water at room temperature until the membrane detached from the glass. The membranes were then placed between filter papers and air-dried under a glass plate.

### 2.4. Hydrothermal Reduction of GO/PAN Membranes

The first stage of the research of in situ hydrothermal graphairene oxide reduction was carried out for composite membrane D, i.e., that containing the highest addition of graphene oxide (2% GO/PAN).

For this purpose, successive membranes were placed between two rubber gaskets (a flat disks with an external diameter of 15 cm and an internal diameter of 10 cm) and then placed into a pressure vessel equipped with a thermometer, a manometer and a safety valve. The vessel was filled with distilled water to a level so that the membrane did not touch the water surface, then it was tightly closed and heated until boiling (temperature: 120 °C, pressure: 0.2 MPa). Boiling was maintained for 30, 45 and 60 min. Hydrothermally reduced composite membranes (D30, D45, D60) were sandwiched between filter papers and dried at room temperature under load.

**Figure 2:**
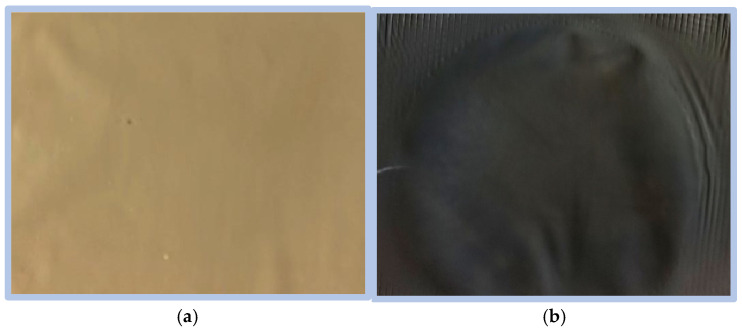
Photograph of 2% GO/PAN membranes: before (**a**) and after hydrothermal reduction (**b**).

As a result of the experiment, it was observed that after hydrothermal reduction an identical color change in composite membranes (D30, D45, D60) occurred from light brown to gray (Figure 2). We observed a similar GO-related color change in our previous work, in which the reduction of GO to rGO was carried out in dimethyl phthalate [10].

Then, under the same pressure and temperature conditions for 60 min, the hydrothermal reduction process was carried out for the remaining membranes: A (from pure PAN), B and C (containing 0.5% GO/PAN and 1% GO/PAN, respectively).

### 2.5. General Characteristics of Membranes

The thickness (*l*) of the membranes was measured with an Elmetron MG-1 thickness gauge. Samples with a diameter of 7.6 cm were weighed using a Sartorius CP224S-0CE analytical balance (Sartorius, Göttingen, Germany) with an accuracy of 0.0001 g.

The mass per unit area (*W_s_*) (g/cm^2^) and the apparent density (*d_m_*) (g/cm^3^) of the membranes were calculated using Formulas (1) and (2)

(1) $$W_{s}=\frac{w}{s}$$

(2) $$d_{m}=\frac{w}{s\times l}$$

where *w*—mass of a membrane with a diameter of 7.6 cm, *s*—membrane surface area (cm^2^) and *l*—membrane thickness (cm).

Water content (U) was determined using a RADWAG MA 110.R moisture analyzer (RADWAG Balances and Scales, Radom, Poland). To perform the measurement, the membranes were placed in distilled water for 24 h. The membranes were then filtered using filter paper and dried in a moisture analyzer in three steps. First, they were dried for 3 min at 30 °C, then for 3 min at 45 °C and finally at 60 °C until a constant weight was obtained.

The static contact angle was measured using a goniometer (FIBRO System AB PG-1, Testing Machines, Inc., New Castle, DE, USA); thus the tests were made in the skin (top) layer of the membranes.

### 2.6. Measurements of Water Flux

The transport properties of the formed membranes were tested using a Millipore Amicon 8400 ultrafiltration cell (Merck, Darmstadt, Germany) with a capacity of 350 cm^3^ and a 7.6 cm membrane diameter which was equipped with an equalizing tank with a capacity of 800 cm^3^. First, dry membranes were immersed in distilled water for 1 h. Then, they were treated with distilled water for additional 2 h under a pressure of 0.2 MPa to improve membrane stability. Ultrafiltration (UF) tests were performed at operational pressures of 0.1, 0.15 or 0.2 MPa under room temperature conditions. Permeate flux (*J_v_*) was calculated using Formula (3)

(3) $$J_{v}=\frac{Q}{A\times t}$$

where *J_v_* is water flux (dm^3^/m^2^ × h), *Q* is the permeate volume (L), *A* is the effective membrane area (m^2^) and *t* is the permeation time (h).

### 2.7. Characterization Techniques

The prepared composite membranes were characterized using Fourier Transform Infrared spectroscopy (FTIR), Raman spectroscopy, X-ray diffraction (XRD), thermogravimetric analyses (TGAs) and scanning electron microscopy (SEM).

All measurements were performed using a Nicolet 6700 FT-IR spectrometer (Thermo Electron Corp., Madison, WI, USA) equipped with an MTEC model 300 photoacoustic accessory. Samples for photoacoustic testing were placed in a special snap holder. The following measurement parameters were used: resolution, 4 cm^−1^; spectral range, 500–4000 cm^−1^; detector—deuterated thioglycine (DTGS); number of scans, 64. Data collection and post-processing were performed using OMNIC software (v. 8.0, Thermo Electron Corp.).

Raman spectroscopy was conducted using a Renishaw InVia micro-Raman spectrometer (Renishaw plc, Wotton-under-Edge, UK), with an excitation laser at 633 nm, a laser power of approximately 15 mW, a spectral resolution of 2 cm^−1^, and a long working distance objective Olympus LMPLFLN 20× (Evident, Hamburg, Germany).

Thermal properties of membranes were studied by thermogravimetric (TGA). The tests were studied using a TA Instruments Q500 (USA) analyzer (TA Instruments, New Castle, DE, USA). Samples were heated from 30 °C to 550 °C at a rate of 20 °C/min under a nitrogen atmosphere (flow rate: 60 cm^3^/min). TGA data were processed and interpreted using Universal V4.5A software (TA Instruments).

The supermolecular structure of membranes was determined by means of the wide-angle (WAXS) and small-angle (SAXS) X-ray scattering methods. WAXS investigations were performed with a URD-65 Seifert (Germany) diffractometer (Seifert Systems GmbH, Radevormwald, Germany). SAXS experiments were carried out by means of an MBraun camera (M. Braun Inertgas-Systeme GmbH, Garching, Germany) utilizing a conventional Kratky collimation system. In both methods CuKα radiation was used. The crystallinity of the membranes was evaluated based on WAXS measurements. For this purpose, each WAXS curve was deconvoluted into crystalline and amorphous scattering components using the WaxsFit [36] profile fitting software. The crystallinity index was calculated as a ratio of the area under crystalline peaks attributed to PAN to the total area of the scattering curve. The pore volume fraction of membranes was estimated using the SAXS method. For this purpose, assuming a PAN density equal to 1.163 g/cm^3^ [37], the electron density of this polymer was determined at the level of 370 electrons/nm^3^. Next, the calculations were carried out in accordance with the methodology described earlier in [38]. The studies characterize the content of pores on length scales from 1 nm to 60 nm, according to the resolution of the SAXS equipment used.

Cross-section and surface porosities and membrane surface pore size were analyzed using a Phenom ProX scanning electron microscope (SEM) manufactured by Thermo Fisher Scientific (Eindhoven, The Netherlands) operating at 10 kV. Samples were frozen in liquid nitrogen and fractured to obtain cross-sections. The diffusion method was utilized to spray a 5 nm thick layer of gold to all the membrane samples using a LEICA ACE 200 low-vacuum sprayer (Pik Instruments, Piaseczno, Poland). At least three membrane pieces and three images of each piece were measured to obtain one data point. The membrane surface porosity was measured based on the surface images using PoroMetric software developed by PhenomWorld.

## 3. Results and Discussion

Based on the literature analysis, an attempt was made to hydrothermally reduce GO in the PAN matrix under mild conditions: at a temperature of 120 °C and a pressure of 0.2 MPa, with the reduction time being selected experimentally (0, 30, 45, 60 min). The structural studies—FTIR, Raman, WAXS and TGA—allowed us to follow the in situ reduction process of composite membranes containing the largest addition of graphene oxide (2% GO relative to the PAN matrix).

### 3.1. FTIR Analysis

**Figure 3:**
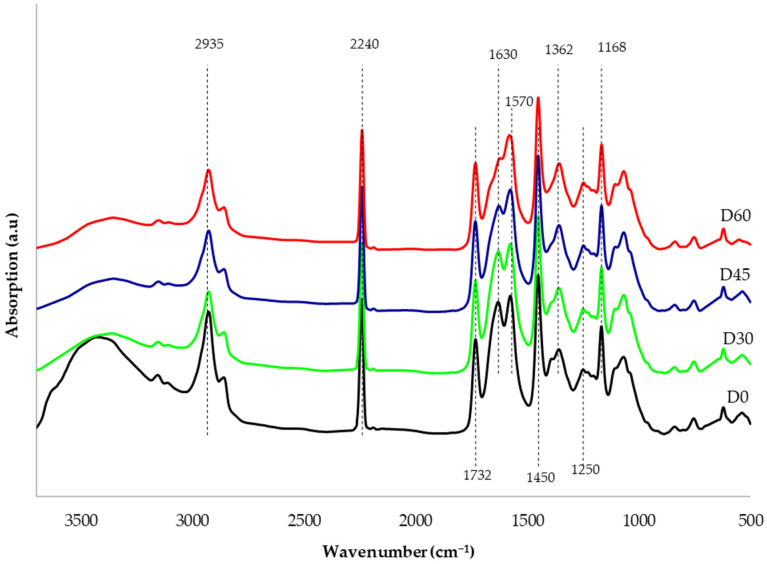
FTIR spectra of composite membranes subjected to hydrothermal reduction process (D0, D30, D45, D60).

In the FTIR spectra (Figure 3), characteristic absorption bands present in PAN can be observed: at 2935 cm^−1^, there are C-H group stretching vibrations; at 2240 cm^−1^, there are C≡N group stretching vibrations; while at 1450 cm^−1^ and 1362 cm^−1^, deformation vibrations in CH_2_ and CH groups are apparent [39]. The band at around 1732 cm^−1^ is characteristic of the stretching vibrations of the carboxyl group -COOH, occurring both in PAN (a copolymer containing about 6% of ester) as well as in GO and rGO [23]. The absorption band at 1168 cm^−1^ comes from the C-O bond, whereas the band at 1250 cm^−1^ is characteristic of the C=O bond. Both peaks originate from rGO, the amount of which—as can be observed—increases slightly in the D30, D45, and D60 membranes. The spectra also include bands at 1630 cm^−1^ and 1570 cm^−1^, which originate from the vibrations of the C=C bond [23,40]. The band at 1250 cm^−1^, on the other hand, comes from the stretching vibrations of C=O in alkoxy and epoxy groups [3]. The strong, broad peak in the range of 3000–3600 cm^−1^ results from the vibration of –OH groups originating from GO (D0 membrane). This peak gradually disappears during hydrothermal reduction (D30, D45, D60), which may indicate the formation of rGO [3].

### 3.2. Raman Spectroscopy Analysis

In Raman spectroscopy studies, the D and G bands, which can be observed on the curves, are attributed to the stretching bonds at sp2 carbon and out-of-plane vibrations near structural distortions (edges, vacancies, oxide groups, etc.), respectively [34]. The ratio of the D and G peak intensities (ID/IG) reflects the degree of disordering in the graphene flakes, and higher ID/IG values correspond to a greater number of defects [41].

**Figure 4:**
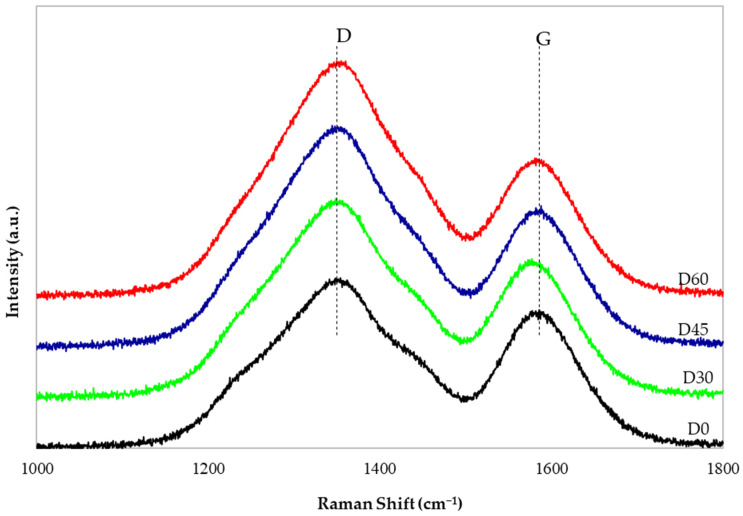
Raman spectra of composite membranes subjected to hydrothermal reduction process (D0, D30, D45, D60).

Figure 4 presents the Raman curves of the composite membranes before (D0) and after the hydrothermal reduction process (D30, D45, D60). All samples exhibit a D peak at approximately 1070 cm^−1^ and a G peak at approximately 1590 cm^−1^. The peak area ratios of the D band and G band (ID/IG) for the D0, D30, D45 and D60 membranes are, respectively, as follows: 2.17; 2.56; 2.76; and 2.93. The increasing ID/IG values result from the greater number of defects formed during the increasingly longer time of GO reduction to rGO. These defects arise due to the removal of oxygen functional groups and a reduction in the average size of the structures forming rGO [23]. This phenomenon is particularly evident in the FTIR spectra (Figure 3), where a clear decrease in the broad peak originating from hydroxyl group vibrations can be observed. It is also observed in Figure 4 that the ratio of the peak heights of the D band to the G band (D:G) increases with the extension of the hydrothermal reduction time and is as follows: 1.23; 1.44; 1.57; and 1.70 (for D0, D30, D45, D60 membranes, respectively). Our research has shown that the reduction of GO/PAN composite membranes causes defects resulting from the formation of structures derived from rGO, with the highest number of these defects observed in the D60 membrane. The results obtained using Raman spectroscopy confirm that the changes observed in the FTIR spectra (Figure 3) are associated with the progressive reduction of GO to rGO.

### 3.3. WAXS Analysis

**Figure 5:**
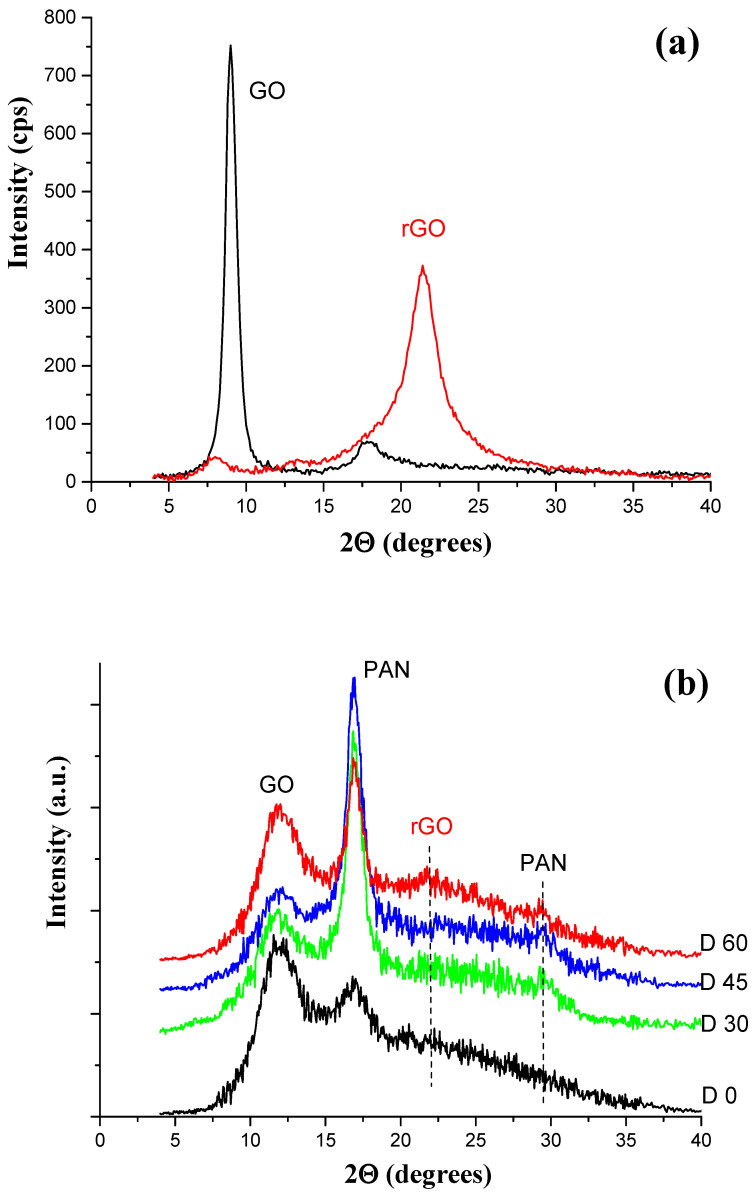
WAXS patterns of (**a**) graphene oxide (GO) and reduced graphene oxide (rGO); (**b**) GO/PAN (D0) and rGO/PAN (D30, D45, D60) membranes.

Figure 5a shows the WAXS patterns of the graphene oxide (GO) and the reduced graphene oxide (rGO) to correctly interpret the diffraction patterns of the tested membranes. For GO, a strong sharp peak at 2θ ≈ 9°, corresponding to an interlayer spacing of 1 nm, is observed (the second-order peak of this structure at 2θ ≈ 18° is also visible). During thermal treatment, the oxygen-containing groups of GO rapidly decompose, causing disintegration of GO aggregates and the formation of rGO aggregates, in which the distance between graphene layers decreases to 0.4 nm. As a result, the diffraction peak of the rGO structure is observed at 2θ ≈ 21.5°. In case the GO reduction is not complete, the first-order peak of GO structure is still observed on the WAXS curve.

The diffraction pattern of the D0 membrane (Figure 5b) shows a diffraction maximum at an angle of 2θ ≈ 10.7°, characteristic of the layered structure of GO, and a weak peak at an angle of 2θ ≈ 17°, corresponding to the diffraction plane (100) characteristic of the pseudocrystalline structure of PAN [32,37]. During thermal treatment of the membranes, the structure of both GO and PAN changes, which can be observed in the WAXS curves for D30–D60 membranes (Figure 5b).

The intensity of the GO peak decreases slightly, suggesting the ongoing GO reduction process. However, because the angular position of the rGO peak is similar to that of the amorphous PAN halo, the peak is masked by this halo and is not visible on the curves for D30 and D45 membranes. Only when the content of rGO aggregates is sufficiently high can it emerge from the amorphous PAN halo. This is the case for the D60 membrane, whose diffraction pattern already shows it.

Thermal treatment of the membranes also promotes the improvement of the PAN chain ordering, as evidenced by a significant increase in the intensity of the main PAN peak (100) and the appearance of a second less intense peak at 2θ ≈ 29.5°, corresponding to the diffraction plane (110) of this paracrystalline structure. This second effect is especially important, because the appearance of this peak is only possible with good, detect-free ordering. It should be noted that the angular positions of both PAN peaks do not change, which means that the distances between the chains of this polymer are the same in all types of membranes tested. Changes in the supermolecular structure of rGO/PAN membranes also result in changes in the content of PAN-ordered regions in these membranes. For the D0 membrane, the degree of crystallinity is low and amounts to approximately 13%. For the other membranes, its value increases to approximately 20%.

**Figure 6:**
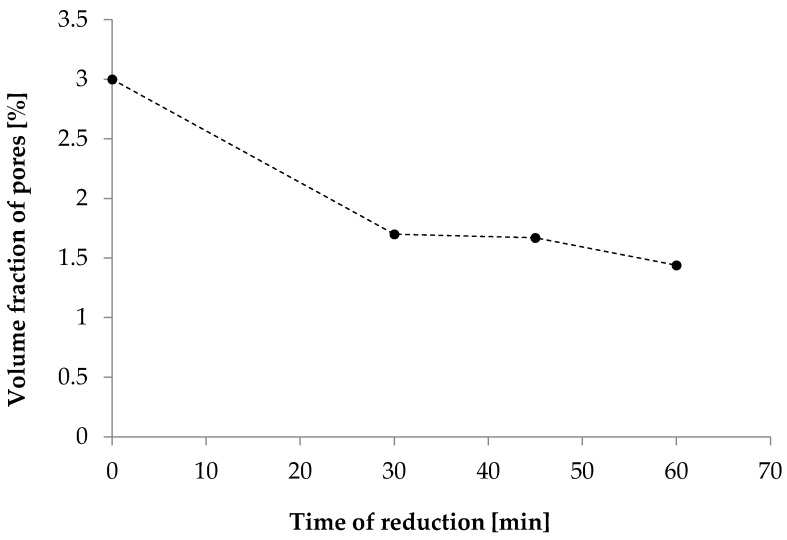
Changes in the nanopore content in the tested membranes.

SAXS studies of the membranes demonstrated that the GO reduction process, the formation of rGO aggregates, and the improvement of PAN chain ordering resulted in a decrease in the content of nanopores with sizes of 1 nm–60 nm compared to the content of pores in the D0 membrane (Figure 6). This effect is the result of either the filling of pores by rGO aggregates or single graphene layers, or the merging of closed nanopores and the formation of more open structures, as shown in SEM images.

### 3.4. TGA

**Figure 7:**
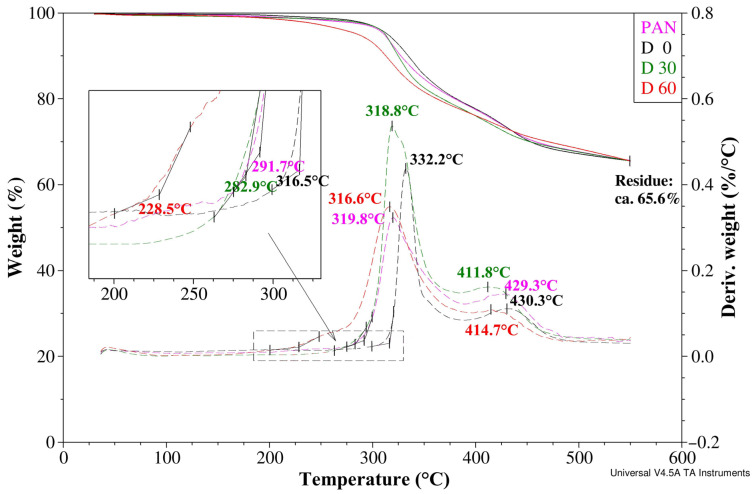
TG and DTG (dashed lines) curves for membrane of pure PAN (pink) and PAN membranes modified with addition of 2% graphene oxide, respectively: D0 (black)—without heat treatment, D30 (green)—heated within 30 min at 120 °C and D60 (red)—heated within 60 min at the same temperature. Black arrow—magnification of the analyzed area.

The thermal properties of the membranes were assessed based on the results of thermogravimetric analyses (Figure 7). The nature of the curves depicting thermal decomposition is analogous for all samples. Thermal decomposition in an oxygen-free atmosphere occurs in a wide temperature range from 220 to 490 °C and is clearly two-stage, as evidenced by two separate peaks on the DTG curves.

The first stage involves the cyclization of PAN chains and the release of low-molecular-weight decomposition products. The onset of thermal dissociation of a membrane of pure PAN is less than 283 °C (extrapolated onset temperature), and the temperature of the highest rate of weight loss in this stage, corresponding to the maximum on the DTG curve, is 319.8 °C. After adding a modifier in the form of 2% by weight of GO (sample D0), both the onset temperature and the maximum rate of mass loss clearly increase to 136.5 °C and 332.2 °C, respectively. Heating of the membranes at 120 °C reversed this pattern. With the increase in heating time, both the initial temperature and the maximum rate of mass loss in the first stage of decomposition decrease monotonically. In the measurement series under consideration, sample D45 (heated at 120 °C for 45 min) was also evaluated. However, its recorded TG and DTG curves were not included in the graph to maintain clarity.

The second stage of decomposition corresponds to the loss of weight of the tested membranes which is associated with further high-temperature restructuring of the carbonized residue. The trend of temperature changes at the highest rate of weight loss, observed in the first stage of decomposition, is maintained (the maximum value of 430.3 °C was recorded for sample D0); however, as the heating time of the modified membranes increases, the temperature does not decrease strictly monotonically. At a temperature of less than 550 °C, the TG curves for the tested membranes become uniform. The carbonized residue remaining at this temperature, consistently for all analyzed samples, is approximately 65.6%. Based on literature reports [31,42] it can be assumed that this residue consists of aromatic carbon rings with significantly ordered structure.

The consistency and complementarity of the structural WAXS and TGA results presented in this article should be noted in a much broader context. The addition of 2% *w*/*w* of GO modifier reduces the ordering of the supermolecular structure of the PAN matrix, which is manifested by a decrease in the intensity of diffraction maximum at an angle of 2θ ≈ 17° (D0 membrane). Similarly, in the case of the discussed membrane, the indicated additive causes a shift in the onset of decomposition by almost as much as 25 °C toward higher temperatures. In the case under consideration, the first stage of thermal dissociation corresponds to the cyclization of PAN chains, accompanied by the release of low-molecular-weight gaseous (decomposition) products. This is clearly demonstrated by a significant sample weight loss and the appearance of a maximum in the DTG curve. Therefore, for the D0 membrane, such a significant shift in the above-mentioned effects towards higher temperatures clearly indicates difficulties in the ordering of the structure at the supermolecular level. This trend is reversed after heat treatment of the modifier. We can observe the conversion of GO to rGO, expressed by a reduction in the intensity of the diffraction maximum at an angle of 2θ ≈ 10.7°. The resulting reduced graphene oxide, therefore, becomes an effective promoter of ordering of the PAN matrix structure. This ordering, as shown above, is inextricably linked to chain cyclization, removal, and release of peripheral groups, which constitute gaseous decomposition products. The greater the ease (mobility) of structure ordering, the greater the effect of this ordering, which is directly related to the weight loss observed in the TG and DTG curves at lower temperatures. For the discussed series of membranes, this is expressed by a reduction in the onset of decomposition (corresponding to cyclization of PAN chains) by up to 88 °C for the D60 sample compared to the membrane that was not heat-treated.

### 3.5. Physicochemical and Transport Properties of Membranes

**Figure 8:**
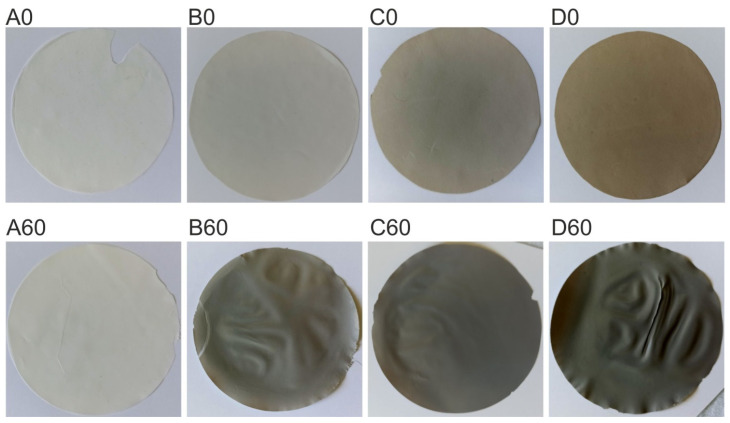
Photographs of membranes before (A0, B0, C0, D0) and after the hydrothermal reduction process (A60, B60, C60, D60).

The structural studies indicated that the optimal time of hydrothermal reduction of GO to rGO in the polymer matrix is 60 min. Therefore, the next step was to reduce the composite membranes containing 0%, 0.5% and 1% GO/PAN (A0, B0, C0), obtaining A60, B60 and C60 membranes (Figure 8).

In the photographs (Figure 8) the difference in the color of the composite membranes can be seen with the naked eye. Pure PAN membranes (A0 and A60) are white, both before and after reduction. The B0, C0 and D0 composite membranes are characterized by a very light gray-brown color, increasing in saturation with the increase in GO content (in the order of 0.5; 1.0 to 2.0% of GO/PAN). However, after the hydrothermal reduction process, the B60, C60 and D60 membranes (due to the presence of rGO) are noticeably darker compared to the non-reduced membranes.

Then, selected physical properties (thickness, apparent density, water sorption, contact angle) and transport properties were tested.

**Figure 9:**
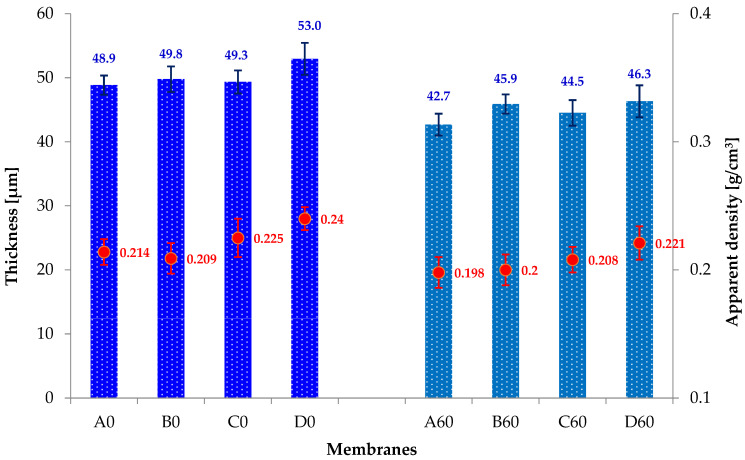
Thickness (blue bars on the chart; black lines—standard deviations) and apparent density (red points on the chart; red lines—standard deviations) of membranes before (A0, B0, C0, D0) and after hydrothermal reduction (A60, B60, C60).

Analyzing Figure 9, it can be observed that the addition of GO slightly affects the thickness of composite membranes A0, B0, C0 and D0. The D0 membrane is approximately 8% thicker compared to the A0 membrane (pure PAN). However, when comparing the series of reduced membranes (A60, B60, C60, D60) to the membranes before reduction, it is observed that after the reduction process the thickness of the membranes is lower by 16% (A60) and 8; 11; 16% (for the B60, C60, D60 membranes, respectively).

We obtained interesting results in studies of the apparent density of composite membranes. In Figure 9, it is observed that the apparent density values of the membranes B0, C0 and D0 slightly increase with the increase in GO content. However, after the hydrothermal reduction process, the apparent density values are slightly lower (A60, B60, C60, D60) than for the A0, B0, C0 and D0 membranes.

Therefore, it can be concluded that the hydrothermal reduction process causes a slight decrease in the thickness and apparent density of all tested membranes.

**Figure 10:**
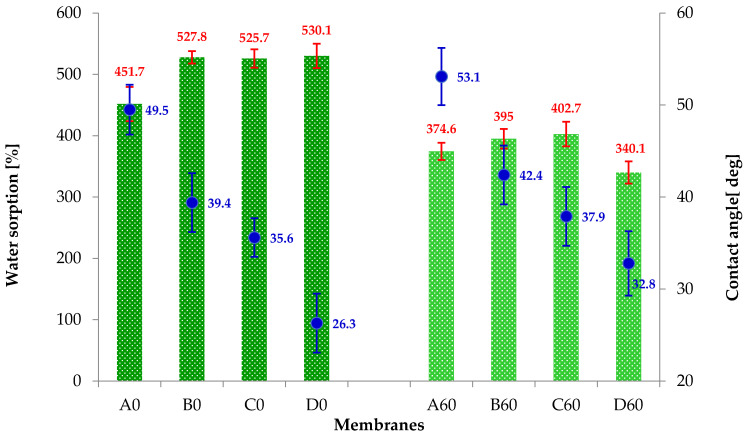
Water sorption (green bars on the chart; red lines—standard deviations) and contact angle (blue points on the chart, blue lines—standard deviations) of membranes before (A0, B0, C0, D0) and after hydrothermal reduction (A60, B60, C60).

Water sorption studies (Figure 10) demonstrated that the pure PAN membrane (A0) absorbs about 15% less water than the composite membranes. B0, C0 and D0 membranes are characterized by water sorption of approximately 530%. Analyzing the graph above, it can also be seen that the hydrothermal reduction process clearly reduces the sorption properties of all tested membranes by 17, 25, 24 and 36% (for A60, B60, C60 and D60, respectively).

The results presented in Figure 10 confirm that the increase in the amount of nanoadditive (GO and rGO) results in a decrease in the contact angle values. Samples after hydrothermal reduction are characterized by higher contact angle values than the non-reduced membranes (A0, B0, C0, D0). This may indicate that during the hydrothermal treatment, the PAN structure (A60) is ordered and rGO structures are formed in the PAN matrix (B60, C60, D60).

Therefore, GO imparts hydrophilic properties to GO/PAN composite membranes, and the hydrothermal reduction process results in a decrease in water sorption and an increase in the contact angle, which indicates the presence of hydrophobic rGO in the membranes (B60, C60, D60). Moreover, the study of physicochemical properties confirms the results obtained using WAXS (Figure 5b), which show that thermal treatment of the membranes also promotes the improvement of the PAN chain ordering.

**Figure 11:**
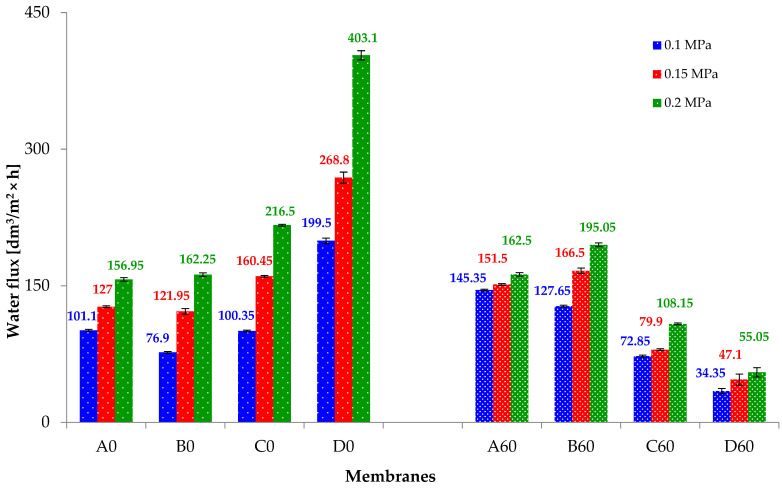
Transport properties of membranes before (A0, B0, C0, D0) and after hydrothermal reduction (A60, B60, C60).

The results of the tests on the transport properties of the membranes (before and after hydrothermal reduction) are summarized in Figure 11. Analyzing the results obtained for membranes A0, B0, C0 and D0, it can be observed that the increase in operating pressure results in the increase in water flux through subsequent membranes. In the case of a PAN membrane (A0), the water flux takes the values of ~100; 127; 157 [dm^3/^m^2^ × h] for the following respective working pressures: 0.1, 0.15 and 0.2 MPa. In general, composite membranes are characterized by an increase in water flux rates. For a working pressure of 0.2 MPa, the water flux through the B0, C0 and D0 membranes is ~162; 217; 403 [dm^3/^m^2^ × h]. It can therefore be concluded that the water flux through the composite membranes increases with the increase in the content of GO nanoadditive in the PAN matrix.

The results of the tests on the transport properties of the membranes after the hydrothermal reduction process are summarized in Figure 11. Pure PAN membranes (reference samples) were also subjected to a heating process in steam at a temperature of 120 °C and a pressure of 0.2 MPa. It was surprising to note that the water flux rates through the A60 membranes were similar and amounted to ~145, 152 and 163 [dm^3/^m^2^ × h]. The obtained results may suggest that the conditions of the process (hydrothermal treatment) could have influenced the ordering of the internal structure of PAN, which is confirmed by WAXS studies.

Tests of the transport properties of C60 and D60 composite membranes, on the other hand, showed that the reduced nanoadditive resulted in a decrease in water flux by ~50% and even ~86%, which, for the working pressure of 0.2 MPa, was ~108; 55 [dm^3/^m^2^ × h]. Therefore, it can be concluded that during hydrothermal treatment of composite membranes, the process of GO reduction to rGO takes place, which was also confirmed by WAXS studies (Figure 5b).

### 3.6. SEM Analysis

The physicochemical and transport properties of the membranes obtained in the experiment result directly from their internal structure and the selective layer. Both properties can be determined using scanning electron microscopy (SEM).

**Figure 12:**
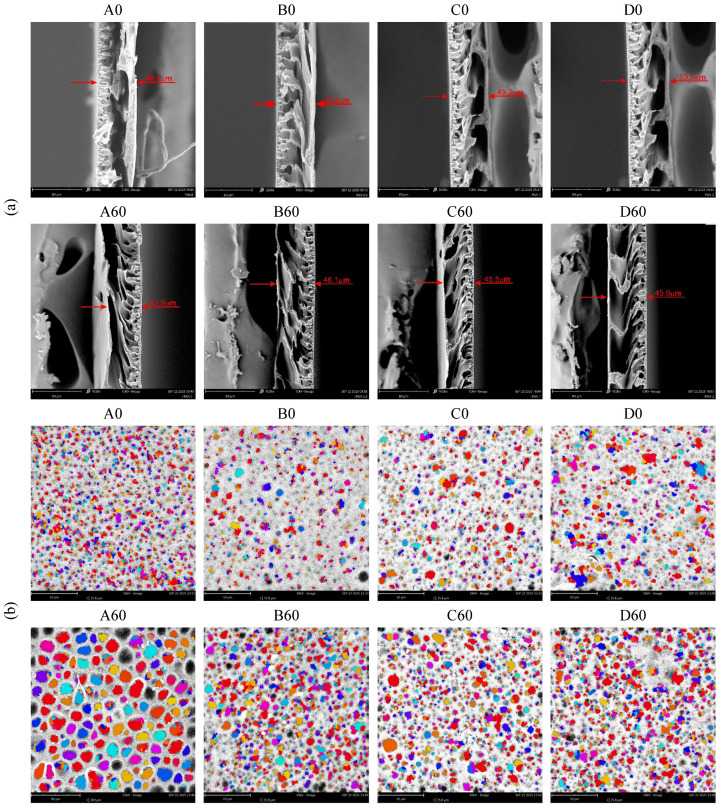
SEM images of the membrane before (A0, B0, C0, D0) and after the hydrothermal reduction process (A60, B60, C60, D60): (**a**) cross-section (magnification of 1000×; red arrows indicate the membrane thickness); (**b**) pore distribution in the skin layer obtained using PoroMetric software (magnification of 10,000×).

SEM images (Figure 12a) of cross-sections show that all obtained membranes have a layered structure, which is characteristic of asymmetric membranes. The skin layer of the membranes is approximately 2.0–5.0 µm thick and has a characteristic shape—fingerprint-like. In the supporting layer, large chambers composed of very porous walls are observed. The SEM images do not show any clear differences in the structure of the composite membranes, both before (A0, B0, C0, D0) and after the hydrothermal reduction process (A60, B60, C60, D60). When comparing the thickness results obtained using the thickness gauge (Figure 9) and those read from the SEM images (Figure 12a), significant similarities can be observed. The SEM images of membranes before reduction showed that they are slightly thicker than after reduction (A60, B60, C60, D60). Therefore, both methods provide comparable results.

Images of the skin layer of the membranes taken using PoroMetric software (Figure 12b) enabled the analysis of their porosity. Already at first glance it is apparent that after hydrothermal treatment, the A60, B60, C60 and D60 membranes are characterized by a higher number of pores (or depressions on the surface) and their larger sizes, compared to the A0, B0, C0 and D0 membranes.

**Figure 13:**
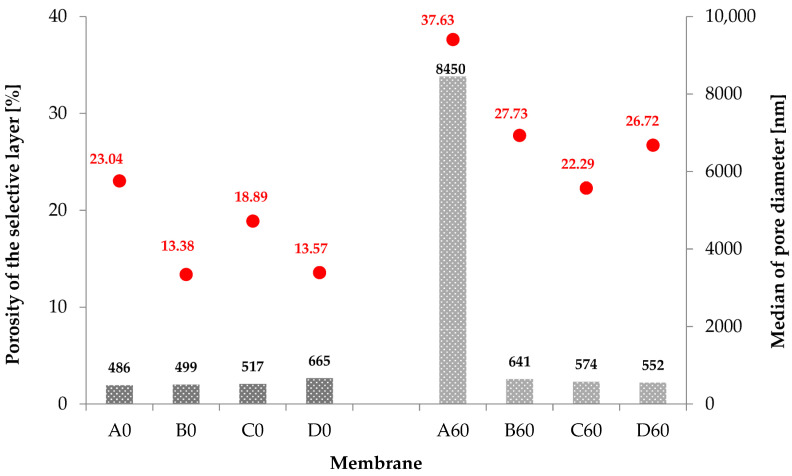
Porosity (red points on the chart) of the selective layer and median pore diameter (gray bars on the chart) of the membranes: before (A0, B0, C0, D0) and after hydrothermal reduction process (A60, B60, C60, D60) determined using PoroMetric software.

The analysis based on PoroMetric software allowed for determination of the porosity of the selective layer and the determination of the median pore size for each membrane (Figure 13). The tests of the A0, B0 and C0 membranes demonstrate that they are characterized by similar median pore size values in the selective layer, which are 486, 499 and 517 nm, respectively. The D0 membrane, on the other hand, has 665 nm pores. The pore content of the selective layer of the B0, C0 and D0 membranes was 13, 19 and 14%, respectively, which means that the pores occupied less than 20% of the surface of the skin layer. Comparing the porosity test results for the A0, B0, C0 and D0 membranes, it can be concluded that the transport properties of these membranes also tend to increase (Figure 11).

Figure 13 also presents the results of the selective layer porosity tests for the A60, B60, C60 and D60 membranes. The pores appearing on the skin layer after the hydrothermal reduction process constitute ~28, 22 and 27% of the total surface of the B60, C60 and D60 membranes. Their median size, slightly higher than before reduction, is 641, 574 and 552 nm, respectively. By comparing the porosity tests with the water flux rate through the tested membranes (B60, C60, D60), it can be concluded that the process of hydrothermal reduction of GO to rGO causes profound changes in the composite material. As a result of the changes occurring inside the membranes, their transport properties decrease.

However, the pure PAN membrane behaves differently. In this membrane heat treatment causes a large increase in the number of pores in the selective layer (~38%). The median pore size for sample A60 was over 15% larger than before reduction. Comparing the porosity results with the transport properties of the A60 membrane, it can be concluded that during the heating process, the internal structure of PAN is ordered, as confirmed by WAXS studies.

The research also showed that the use of the SEM method and PoroMetric software does not allow for the identification of active pores that directly participate in separation processes.

Therefore, it can be inferred that the transport properties of membranes subjected to the hydrothermal reduction process are closely related to the physical and chemical processes occurring within the membrane structure. As a result of the reduction carried out at a temperature of 120 °C, a pressure of 0.2 MPa, and in the presence of water vapor, there is an increase in the contact angle (hydrophobization), a decrease in thickness, a decrease in apparent density, and a reduction in water sorption.

## 4. Conclusions

This paper investigated the use of a method of in situ hydrothermal reduction of GO to rGO in bulk, which has not been described in the literature so far. The membrane reduction was carried out under mild conditions: in a steam atmosphere, at a temperature of 120 °C, and under a pressure of 0.2 MPa. Structural studies clearly confirmed that during 60 min of heating of GO/PAN composite membrane samples (B0, C0, D0), GO was reduced to rGO, resulting in the formation of rGO/PAN composite membranes (B60, C60, D60). In the FTIR curves, the disappearance of characteristic peaks originating from GO and the simultaneous formation of peaks originating from rGO were observed. The formation of defects characteristic of the rGO structure formed during hydrothermal reduction was observed in the Raman spectra. WAXS curves showed GO reduction and ordering of the internal structure of PAN.

Moreover, the in situ hydrothermal reduction process of the composite membranes clearly influenced the physicochemical properties: a decrease in thickness, a reduction in apparent density and water sorption, and an increase in the contact angle. During this process, chemical changes (reduction of GO to rGO) and physical changes in the membrane structure occurred, which influenced their transport properties (reduction in water flux by ~50% and even ~86% compared to the initial membranes) of the B60, C60 and D60 membranes.

The studies described above indicate the possibility of conducting in situ hydrothermal reduction of GO in the polymer bulk. The method presented in this paper, which is inexpensive, simple and environmentally friendly, may be an alternative to obtaining composites containing rGO nanoadditive.

## Figures and Tables

**Table 1:** Composition of membrane-forming solutions.

Membrane Designation	Type of Membrane	Amount of 3% GO/DMF [g]	Amount of 12% PAN/DMF [g]	Conc. of GO [% *w*/*w*]	Conc. of PAN [% *w*/*w*]
**A**	PAN	0	50	0	100.0
**B**	0.5% GO/PAN	1	50	0.5	99.5
**C**	1% GO/PAN	2	50	1.0	99.0
**D**	2% GO/PAN	4.06	50	2.0	98.0

## Data Availability

The original contributions presented in this study are included in the article. Further inquiries can be directed to the corresponding author.
